# Association Between Glycemic Gap and In-hospital Outcomes in Aneurysmal Subarachnoid Hemorrhage

**DOI:** 10.3389/fneur.2021.714341

**Published:** 2021-11-23

**Authors:** Philip Y. Sun, Roy A. Poblete, Peggy L. Nguyen, Sebina F. Bulic, May A. Kim-Tenser, Jonathan Marehbian, Steven Y. Cen, Benjamin A. Emanuel

**Affiliations:** ^1^Department of Neurology, Los Angeles County + University of Southern California Medical Center, Los Angeles, CA, United States; ^2^Department of Neurology, Keck School of Medicine, University of Southern California, Los Angeles, CA, United States

**Keywords:** aneurysmal subarachnoid hemorrhage, hyperglycemia, glycemic gap, outcomes, mortality

## Abstract

**Introduction:** Glycemic gap (GG), as determined by the difference between glucose and the hemoglobin A1c (HbA1c)-derived estimated average glucose (eAG), is associated with poor outcomes in various clinical settings. There is a paucity of data describing GG and outcomes after aneurysmal subarachnoid hemorrhage (aSAH). Our main objectives were to evaluate the association of admission glycemic gap (aGG) with in-hospital mortality and with poor composite outcome and to compare aGG's predictive value to admission serum glucose. Secondary outcomes were the associations between aGG and neurologic complications including vasospasm and delayed cerebral ischemia following aSAH.

**Methods:** We retrospectively reviewed 119 adult patients with aSAH admitted to a single tertiary care neuroscience ICU. Spearman method was used for correlation for non-normality of data. Area under the curve (AUC) for Receiver Operating Characteristic (ROC) curve was used to estimate prediction accuracy of aGG and admission glucose on outcome measures. Multivariable analyses were conducted to assess the value of aGG in predicting in-hospital poor composite outcome and death.

**Results:** Elevated aGG at or above 30 mg/dL was identified in 79 (66.4%) of patients. Vasospasm was not associated with the elevated aGG. Admission GG correlated with admission serum glucose (*r* = 0.94, *p* < 0.01), lactate (*r* = 0.41, *p* < 0.01), procalcitonin (*r* = 0.38, *p* < 0.01), and Hunt and Hess score (*r* = 0.51, *p* < 0.01), but not with HbA1c (*r* = 0.02, *p* = 0.82). Compared to admission glucose, aGG had a statistically significantly improved accuracy in predicting inpatient mortality (AUC mean ± SEM: 0.77 ± 0.05 vs. 0.72 ± 0.06, *p* = 0.03) and trended toward statistically improved accuracy in predicting poor composite outcome (AUC: 0.69 ± 0.05 vs. 0.66 ± 0.05, *p* = 0.07). When controlling for aSAH severity, aGG was not independently associated with delayed cerebral ischemia, poor composite outcome, and in-hospital mortality.

**Conclusion:** Admission GG was not independently associated with in-hospital mortality or poor outcome in a population of aSAH. An aGG ≥30 mg/dL was common in our population, and further study is needed to fully understand the clinical importance of this biomarker.

## Introduction

Hyperglycemia portends a poor outcome in aneurysmal subarachnoid hemorrhage (aSAH) (1–9). Given its clinical importance, this may be one of several key physiologic derangements that should be targeted to improve patient outcomes. Suggested mechanisms of secondary brain injury promoted by hyperglycemia include cerebral vasospasm, delayed cerebral ischemia (DCI), promotion of an oxidative state, intravascular coagulation abnormalities, cerebral edema promoted by matrix metalloproteinase activity, and metabolic dysfunction (1, 4, 9–11). Serum glucose level alone, however, does not account for a patient's baseline average glucose level.

Glycemic gap (GG) is a measure of an acute derangement in glucose level in response to an active disease state, calculated by the difference between raw capillary glucose level and the estimated average glucose (eAG) derived from serum hemoglobin A1c (HbA1c) level. Recent studies describe its utility in predicting ICU mortality and adverse outcomes among diabetics (12–15); however, these studies included only a minority of patients with a primary neurologic disease. A retrospective study by Yang et al. on acute ischemic stroke patients with diabetes showed that admission glycemic gap (aGG) was superior to admission glucose and HbA1c in predicting worse NIHSS and functional outcome at discharge (16). Another study on aSAH patients used admission glucose to HbA1c ratio to demonstrate that stress-induced hyperglycemia occurred in nearly half of patients and predicted placement of an external ventricular drain (EVD) (17). The utility of GG in aSAH otherwise has not been fully established, especially in its association with hospital course and short-term outcome.

The main study objectives are to evaluate the association of aGG with in-hospital mortality and poor outcome, and to compare the entity's predictive value to that of admission serum glucose in a population of adult patients with aSAH treated at a single center. We hypothesized that elevated aGG is associated with negative patient outcomes and had better predictive accuracy than admission serum glucose level. If true, this would support the use of aGG as an important predictor of outcome in clinical practice and future translational research.

## Methods

### Population and Enrollment

During a period from 2012 to 2018, adult patients admitted to the neurocritical care unit at the academic tertiary care medical center of The University of Southern California with a primary admitting diagnosis of non-traumatic subarachnoid hemorrhage. Patients were excluded from the analysis if (a) subarachnoid hemorrhage was due to a non-aneurysmal cause (e.g., primary intracerebral hemorrhage, dural fistula, trauma), (b) initial clinical exam suggested brain death (bilateral mydriasis with no reactivity to light), or (c) HbA1c was not available. A total of 119 individuals aged ≥18 with a primary diagnosis of aSAH met inclusion and exclusion criteria. Of the 119 patients included in the analysis, 12 had no identified aneurysm on Computed Tomography angiography (CTA) or digital subtraction angiogram (DSA) but distribution of bleed was suggestive of aSAH rather than perimesencephalic SAH.

### Data Collection and Outcome Measures

The institutional review board of The University of Southern California approved the study procedures (HS-16-00265). Patient consent was waived due to the retrospective nature of the study. Data were gathered from the Electronic Medical Record (EMR) and available medical records from transferring hospitals. We extracted information on demographics, presentation, admission serum studies, clinical and radiographic characteristics of aSAH, and hospital course including short-term outcomes. Vasospasm was diagnosed with a combination of transcranial Doppler ultrasound (anterior circulation mean flow velocity > 120 cm/s with a Lindegaard Ratio >3, vertebral or basilar artery mean flow velocity > 80 cm/s), or findings from CTA or DSA. DCI was defined as persistent focal neurologic deficit attributed to vasospasm, or delayed infarct seen on CT or MRI not attributed to the initial bleed or subsequent procedures. Ventilator-free days is an established measure of acute respiratory failure that provides a greater statistical power to treatment effects (18). The entity was defined to be 0 if the patient died before 28 days, the number of days on mechanical ventilation subtracted from 28 if successfully weaned from mechanical ventilation within 28 days, and 0 if the patient required mechanical ventilation for 28 days or more. For those who were discharged within 28 days of admission, it was assumed that they remained free of ventilators after discharge. The patients who did not require an EVD placement during hospitalization were recorded as having the EVD day of 0.

The estimated average glucose (eAG) in the past 3 months was calculated by the standard equation: [(28.7 × HbA1c)-46.7] (mg/dL). Admission glycemic gap was defined as eAG subtracted from admission serum glucose (mg/dL). For dichotomization based on aGG, the cutoff value of ≥30 mg/dL was defined as “elevated.” This cutoff aGG was selected by manually evaluating ROC sensitivity and specificity cutoff values by increments of 10 for in-hospital mortality. This analysis led to selection of 30 mg/dL, which had the sensitivity of 0.90 (95% CI 0.79, 1.0) and specificity of 0.41 (95% CI 0.31, 0.51), demonstrating a high rate of inclusion with reasonable specificity. The primary outcome was the association of admission glucose and aGG with in-hospital mortality and poor composite outcome. Poor composite outcome was defined as any incidence of percutaneous gastrostomy, tracheostomy, discharge to a nursing facility, or hospital death, whereas good composite outcome was absence of these outcomes. This measure serves to identify patients who will require continued nursing care for activities of daily living. This definition has been similarly used in aSAH populations as the Nationwide Inpatient Sample Subarachnoid Hemorrhage Outcome Measure (NIS-SOM) using ICD-9 code-based national administrative data (19). Secondary outcomes included the association of aGG with neurologic complications of DCI and vasospasm.

### Statistical Analysis

Data distribution was examined by histogram and D'Agostino's K-squared test. Spearman method was used for correlation for non-normality of data. Wilcoxon Rank-sum test was used for non-parametric comparisons. If data were normally distributed, mean values with standard deviation (SD) were reported; otherwise, median values with interquartile range (IQR) were reported. Fisher's exact test was used for any subset sample size of below 5. ANOVA was performed for assessment of a global difference in aGG among diabetics (HbA1c of greater than or equal to 6.5), pre-diabetics (HbA1c of 5.7–6.4), and non-diabetics (HbA1c of 5.6 or less). A *post-hoc* contrast test was done to compare aGG in diabetics against pre-diabetics and non-diabetics combined. Area under the curve (AUC) for Receiver Operating Characteristic (ROC) curve was used to estimate prediction accuracy of admission glucose and aGG (Figure 2). A *z*-test was used to compare the AUC between admission glucose and aGG. As previously described, the diagnostic cut-point of aGG at 30 mg/dL was determined by manual assessment of sensitivity and specificity at each 10-unit increment of aGG. Multivariable Poisson regression was used to calculate the relative risk (RR) between the dichotomized GG and inpatient clinical outcomes. Pearson and deviance statistics were used to assess model overdispersion. When model overdispersion was found, a negative binomial model was used instead. We performed a multivariable analysis controlling for known predictors of outcome in aSAH—Hunt and Hess scale score, modified Fisher scale score, and GCS. PASS 2021 (NCSS, LLC) was used for a *post-hoc* power analysis to evaluate the association between aGG and primary outcomes of interest when adjusted for co-variates. All other data analyses were conducted using SAS (version 9.4; SAS Institute Inc, Cary, NC). Statistical testing was performed at an α level of 0.05.

## Results

We identified a total of 119 adult patients with a primary diagnosis of aSAH who met inclusion and exclusion criteria and were included in the analysis. Patient race/ethnicity was as follows: White (*n* = 23), Hispanic (*n* = 56), and others/unknown (*n* = 40). The cohort's mean age was 59 ± 14 years, in whom 33% were men. The overall median Hunt and Hess and modified Fisher grades were 3 and 4, respectively. The overall median aGG was 48 (IQR 20, 89) mg/dL. Table 1 summarizes demographic and clinical admission factors between patients with high (≥30 mg/dL) vs. low (<30 mg/dL) aGG. Our aGG cutoff value of ≥30 mg/dL had the sensitivity of 0.90 (95% CI 0.79, 1.0) and specificity of 0.41 (95% CI 0.31, 0.51) for mortality. For poor composite outcome at the same cutoff value of 30 mg/dL, the sensitivity and specificity were 0.79 (95% CI 0.68, 0.90) and 0.45 (95% CI 0.33, 0.58), respectively. Seventy-nine (66.4%) patients had an elevated aGG of ≥30 mg/dL with the median level of 63 (IQR 48, 108) mg/dL, significantly higher than the lower aGG group with the median level of 10 (IQR −0.5, 20) mg/dL. There was a significantly higher occurrence of comorbid history of diabetes mellitus in the elevated aGG group. Age, sex, race/ethnicity, body mass index (BMI), and prior history of other cardiovascular comorbidities were similar between groups. Median HbA1c did not differ significantly between high vs. low aGG groups. Although ANOVA showed a global difference in the means of aGG among diabetics (86.9 ± 12.9), pre-diabetics (49.2 ± 7.0) and non-diabetics (55.1 ± 6.9) (*p* = 0.04), comparison between the two aGG groups did not indicate heterogeneous distribution (*p* = 0.12). A *post-hoc* contrast test showed a higher aGG in diabetics compared to pre-diabetics and non-diabetics combined (*p* = 0.01). Patients with elevated aGG had a significantly increased admission serum glucose, white blood cell (WBC) count, anion gap, as well as lower bicarbonate. There were similar occurrences of fever and infection on admission.

**Table 1:** Demographic and clinical admission factors between patients with high vs. low admission glycemic gap.

**Variable**	**aGG ≥ 30 mg/dL** **(*n* = 79)**	**aGG < 30 mg/dL** **(*n* = 40)**	**Overall cohort** **(*n* = 119)**	***p*-value^†^**
Age, years (mean ± SD)	58.01 ± 13.04	60.03 ± 14.90	58.69 ± 13.66	0.45
Sex				0.23
Male, *n* (%)	23 (29.1%)	16 (40.0%)	39 (32.8%)	
Female	56 (70.9%)	24 (60.0%)	80 (67.2%)	
Race/ethnicity				0.74
White, *n* (%)	14 (17.7%)	9 (22.5%)	23 (19.3%)	
Hispanic	39 (49.4%)	17 (42.5%)	56 (47.1%)	
Others/unknown^a^	26 (32.9%)	14 (35.0%)	40 (33.6%)	
BMI^b^, kg/m^2^ (median, IQR)	27.46 (23.73, 30.08)	25.96 (23.16, 30.78)	27.22 (23.38, 30.48)	0.47^#^
Diabetes Mellitus based on admission HbA1c^c^				0.13*
Diabetic, *n* (%)	13 (16.5%)	2 (5.0%)	15 (12.6%)	
Pre-diabetic	30 (38.0%)	21 (52.5%)	51 (42.9%)	
Neither	36 (45.6%)	17 (42.5%)	53 (44.5%)	
History of diabetes mellitus				**0.04** *
Yes, *n* (%)	18 (22.8%)	3 (7.5%)	21 (17.7%)	
No	61 (77.2%)	37 (92.5%)	98 (82.4%)	
Coronary artery disease				0.38*
Yes, *n* (%)	3 (3.8%)	3 (7.5%)	6 (5.0%)	
No	76 (96.2%)	37 (92.5%)	113 (95.0%)	
Hypertension				0.07
Yes, *n* (%)	51 (64.6%)	19 (47.5%)	70 (58.8%)	
No	28 (35.4%)	21 (52.5%)	49 (41.2%)	
Chronic renal disease				0.07*
Yes, *n* (%)	6 (7.6%)	0 (0%)	6 (5.0%)	
No	73 (92.4%)	40 (100.0%)	113 (95.0%)	
HFrEF				0.48*
Yes, *n* (%)	2 (2.5%)	2 (5.0%)	4 (3.4%)	
No	77 (97.5%)	38 (95.0%)	115 (96.6%)	
Glasgow Coma Scale (median, IQR)	10 (6, 14)	14.5 (10, 15)	11 (7, 15)	**<0.01** ^#^
Hunt and Hess scale				**<0.01**
1–3 (good), *n* (%)	19 (24.1%)	24 (60.0%)	43 (36.1%)	
4–5 (poor)	60 (76.0%)	16 (40.0%)	76 (63.9%)	
Modified Fisher scale				**<0.01**
0–2 (good), *n* (%)	5 (6.3%)	9 (22.5%)	14 (11.8%)	
3–4 (poor)	74 (93.7%)	31 (77.5%)	105 (88.2%)	
Admission glycemic gap, mg/dL (median, IQR)	63 (48, 108)	10 (−0.5, 20)	48 (20, 89)	**<0.01** ^**#**^
Serum glucose^d^, mg/dL (median, IQR)	191 (165, 224)	124.5 (115.5, 138)	165 (136, 211)	**<0.01** ^**#**^
HbA1c^e^, % (median, IQR)	5.8 (5.5, 6.2)	5.7 (5.5, 6.05)	5.7 (5.5, 6.1)	0.89^#^
Lactate^f^, mmol/L (median, IQR)	2.7 (1.9, 4.25)	1.65 (1.2, 2.9)	2.45 (1.5, 3.8)	**<0.01** ^#^
Procalcitonin^g^, ng/mL (median, IQR)	0.13 (0.1, 0.22)	0.1 (0.07, 0.14)	0.1 (0.1, 0.2)	0.12^#^
Infection^h^				0.1
No abx used, no infection, *n* (%)	8 (10.1%)	10 (25.0%)	18 (15.1%)	
Abx used, no documented infection	38 (48.1%)	16 (40.0%)	54 (45.4%)	
Abx used, documented infection	33 (41.8%)	14 (35.0%)	47 (39.5%)	
Fever				0.71*
Yes, *n* (%)	3 (3.8%)	1 (2.5%)	4 (3.4%)	
No	76 (96.2%)	39 (97.5%)	115 (96.6%)	
Location of aneurysm^i^				0.72
Anterior circulation, *n* (%)	50 (63.3%)	26 (65.0%)	76 (63.9%)	
Posterior circulation	22 (27.9%)	9 (22.5%)	31 (26.1%)	
No clear source	7 (8.9%)	5 (12.5%)	12 (10.1%)	
WBC, 10^3^ cells/μL (median, IQR)	15.57 (10.53, 19.36)	10.28 (8.35, 13.96)	13.8 (9.2, 18.7)	**<0.01** ^#^
Sodium, mmol/L (mean ± SD)	138.52 ± 3.66	138.83 ± 3.84	138.62 ± 3.71	0.67
Bicarbonate, mmol/L (median, IQR)	22 (20, 24)	26 (23.5, 27)	23 (20, 27)	**<0.01** ^#^
Anion gap, mmol/L (median, IQR)	15 (13, 17)	12 (10, 15)	14 (12, 16)	**<0.01** ^#^
Creatinine^j^, mg/dL (median, IQR)	0.80 (0.62, 0.93)	0.70 (0.64, 0.90)	0.79 (0.62, 0.91)	0.25^#^

† *Statistically significant values are given in bold (p < 0.05)*.

a *The patients were unable to or did not provide an answer*.

b *Admission BMI was not available in three patients*.

c *Diabetes is defined as Admission HbA1c of greater than or equal to 6.5, pre-diabetes is defined as HbA1c of 5.7–6.4*.

d *Conversion factor: multiply by 0.0555 to convert glucose from mg/dL to mmol/L*.

e *Conversion factor: multiply by 28.7 and subtract by 46.7 to convert National Glycohemoglobin Standardization Program HbA1c (%) to estimated average serum glucose (mg/dL). Multiply by 0.0555 to convert from mg/dL to mmol/L*.

f *Admission lactate level was obtained in 108 patients*.

g *Admission procalcitonin level was obtained in 57 patients*.

h *Documented infection was determined by obtained cultures and imaging (i.e., chest-ray). Some patients with no documented infection were empirically treated because of suspicion of infection and/or prophylactically for procedures (i.e., external ventricular drain)*.

i *No definitive bleeding source found in 12 patients*.

j *Conversion factor: multiply by 76.25 to convert creatinine from mg/dL to μmol/L*.

# *Wilcoxon Rank-sum test*.

* *Fisher's exact test*.

*SD, standard deviation; BMI, Body Mass Index; IQR, interquartile range; HbA1c, Hemoglobin A1c; HFrEF, heart failure with reduced ejection fraction; WBC, white blood cell count*.

**Figure 1:**
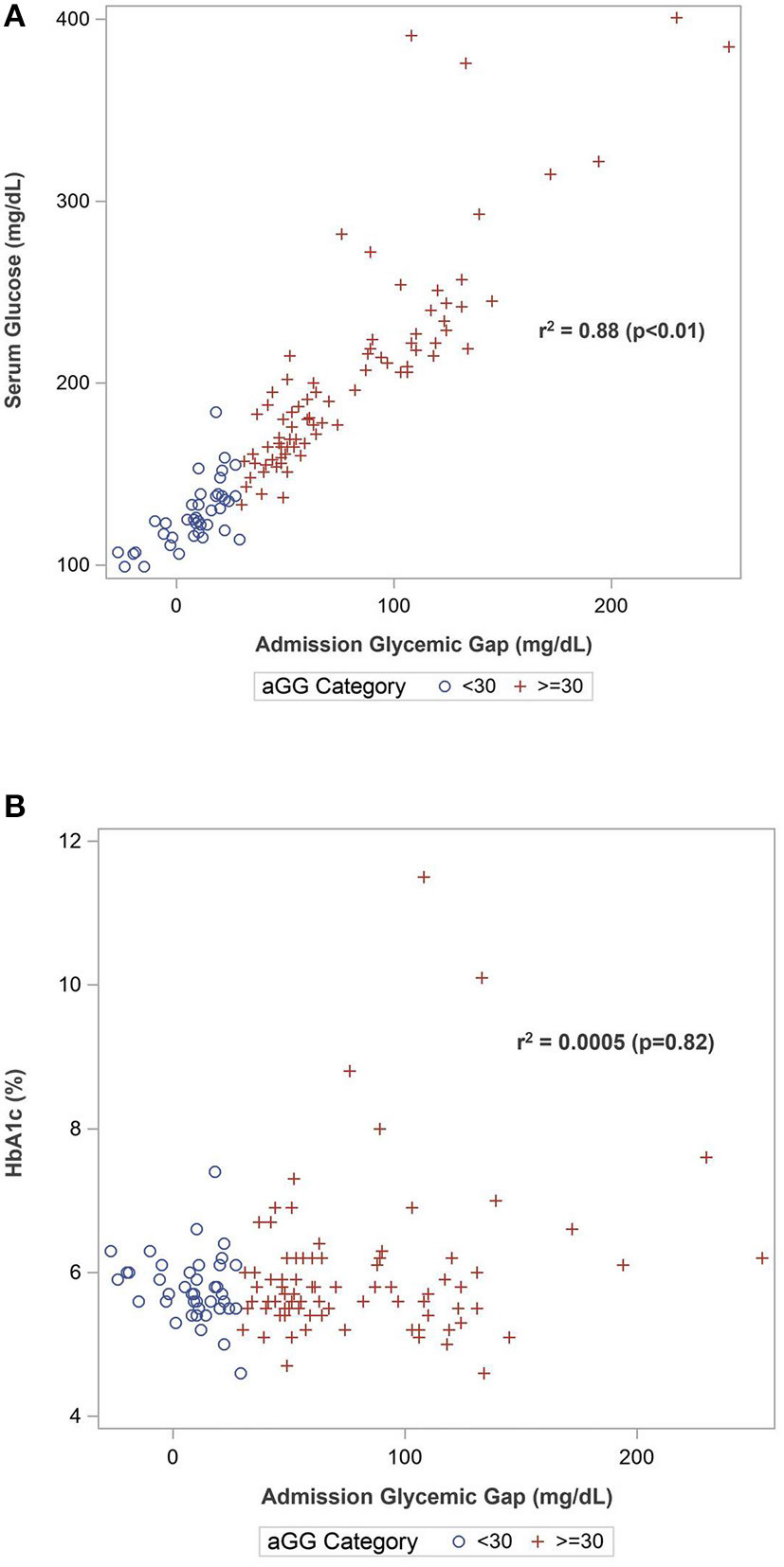
Scatterplots of Spearman's correlation between admission glycemic gap with **(A)** admission serum glucose and **(B)** HbA1c.

Spearman correlation analysis (Table 2) demonstrated that aGG as a continuous variable was strongly correlated with admission glucose (*r* = 0.94, *p* < 0.01) but not with HbA1c (*r* = 0.02, *p* = 0.82). There were positive correlations between aGG and Hunt and Hess scale (*r* = 0.51, *p* < 0.01), modified Fisher scale (*r* = 0.37, *p* < 0.01), lactate (*r* = 0.41, *p* < 0.01), procalcitonin (*r* = 0.38, *p* < 0.01), and WBC on admission (*r* = 0.45, *p* < 0.01). Admission GG was negatively associated with GCS (*r* = −0.50, *p* < 0.01). No correlations were found with regards to age (*r* = −0.04, *p* = 0.66) and BMI (*r* = 0.09, *p* = 0.32). Higher aGG was also inversely correlated with ventilator-free days (*r* = −0.40, *p* < 0.01) and positively correlated with EVD days (*r* = 0.24, *p* < 0.01). Scatterplots demonstrating Spearman correlation between aGG and admission glucose and HbA1c are depicted in Figure 1.

**Table 2:** Spearman correlation between admission glycemic gap and patient characteristics and outcomes.

**Variable**	**Spearman correlation coefficient (*r*)^†^**	***p*-value^†^**
Age, years	−0.04	0.66
BMI (kg/m^2^)^a^	0.09	0.32
Glasgow Coma Scale	−0.50	**<0.01**
Hunt and Hess scale	0.51	**<0.01**
Modified Fisher scale	0.37	**<0.01**
Serum glucose, mg/dL^b^	0.94	**<0.01**
HbA1c, %^c^	0.02	0.82
Lactate, mmol/L^d^	0.41	**<0.01**
Procalcitonin, ng/mL^e^	0.38	**<0.01**
WBC, 10^3^ cells/μL	0.45	**<0.01**
Sodium, mmol/L	−0.03	0.77
Bicarbonate, mmol/L	−0.33	**<0.01**
Anion gap, mmol/L	0.39	**<0.01**
Creatinine, mg/dL^f^	0.14	0.14
Ventilator-free days	−0.40	**<0.01**
EVD days	0.24	**<0.01**
Hospital days	0	0.99
ICU days	0.04	0.67

† *Coefficient (r) values > 0 indicate a positive association; values < 0 indicate a negative association. Statistically significant values are given in bold (p < 0.05)*.

a *Admission BMI was not available in three patients*.

b *Conversion factor: multiply by 0.0555 to convert glucose from mg/dL to mmol/L*.

c *Conversion factor: multiply by 28.7 and subtract by 46.7 to convert National Glycohemoglobin Standardization Program HbA1c (%) to estimated average serum glucose (mg/dL). Multiply by 0.0555 to convert from mg/dL to mmol/L*.

d *Admission lactate level was obtained in 108 patients*.

e *Admission procalcitonin level was obtained in 57 patients*.

f *Conversion factor: multiply by 76.25 to convert creatinine from mg/dL to μmol/L*.

*BMI, Body Mass Index; HbA1c, Hemoglobin A1c; WBC, white blood cell count; EVD, external ventricular drain; ICU, intensive care unit*.

**Figure 2:**
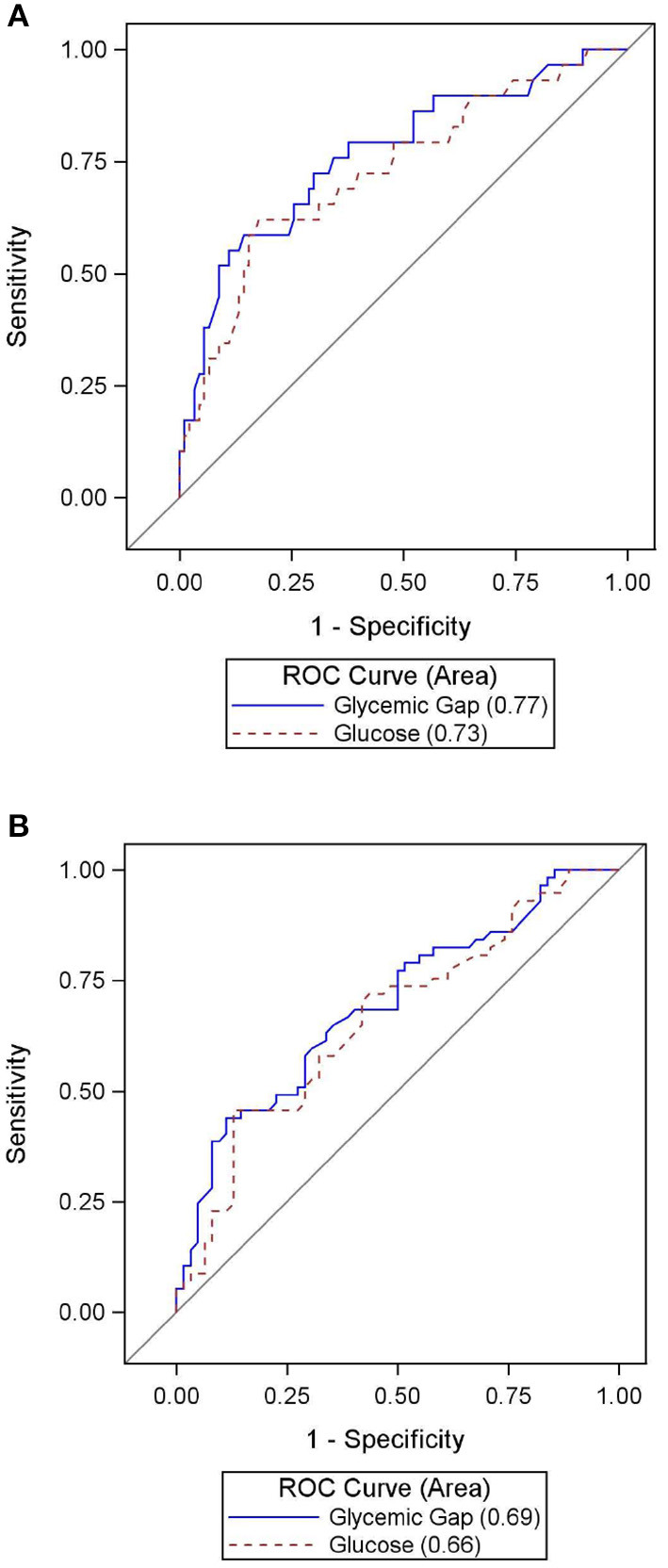
**(A)** Accuracy of admission glucose vs. admission glycemic gap in predicting in-hospital mortality. **(B)** Accuracy of admission glucose vs. admission glycemic gap in predicting poor composite outcome.

Table 3 summarizes hospital outcomes by aGG groups. Patients with elevated aGG ≥30 mg/dL had a greater use of EVDs, but similar length of ICU and hospital stay. While vasospasm was not associated with increased aGG, DCI occurred more commonly among those with higher aGG. Hospital mortality and poor composite outcome both occurred more frequently in those with elevated aGG. As shown in the ROC curves in Figure 2, aGG had a significantly better prediction accuracy than admission glucose in predicting death (AUC mean ± SEM: 0.77 ± 0.05 vs. 0.72 ± 0.06, *p* = 0.03) and a non-significant trend for better accuracy in predicting poor outcome (AUC mean ± SEM: 0.69 ± 0.05 vs. 0.66 ± 0.05, *p* = 0.07). When adjusted for the disease severity measures, aGG was independently associated with EVD days, but not with length of hospitalization, DCI, and vasospasm (Table 4). When unadjusted, the RRs for poor composite outcome and in-hospital mortality were 1.90 (95% CI 1.00, 3.59) and 4.39 (95% CI 1.33, 14.50), respectively, in those with aGG of ≥30 mg/dL. In the multivariable analyses, neither of the outcome measures were statistically significant, with the RRs of 1.07 (95% CI 0.55, 2.09) and 2.10 (95% CI 0.60, 7.30) for poor composite outcome and in-hospital mortality, respectively.

**Table 3:** In-hospital outcomes between patients with elevated vs. non-elevated admission glycemic gap.

**Variable**	**aGG ≥ 30 mg/dL** **(*n* = 79)**	**aGG < 30 mg/dL** **(*n* = 40)**	**Overall cohort** **(*n* = 119)**	***p*-value^†^**
Insulin drip				**<0.01** *
Yes, *n* (%)	21 (26.6%)	2 (5.0%)	23 (19.3%)	
No	58 (73.4%)	38 (95.0%)	96 (80.7%)	
Aneurysmal procedure				0.93*
Clip/wrap, *n* (%)	30 (38.0%)	16 (40.0%)	46 (38.7%)	
Coil	29 (36.7%)	15 (37.5%)	44 (37.0%)	
Others	1 (1.3%)	1 (2.5%)	2 (1.7%)	
None	19 (24.1%)	8 (20.0%)	27 (22.7%)	
Vasospasm				0.17
Yes, *n* (%)	42 (53.2%)	16 (40.0%)	58 (48.7%)	
No	37 (46.8%)	24 (60.0%)	61 (51.3%)	
DCI				**<0.01**
Yes, *n* (%)	30 (38.0%)	5 (12.5%)	35 (29.4%)	
No	49 (62.0%)	35 (87.5%)	84 (70.6%)	
Ventilator-free days (median, IQR)	12 (0, 27)	27 (20, 29)	21 (0, 28)	**<0.01** ^#^
EVD placed?				<0.01
Yes, *n* (%)	73 (92.4%)	25 (62.5%)	98 (82.4%)	
No	6 (7.6%)	15 (37.5%)	21 (17.7%)	
EVD days (median, IQR)	12 (6, 17)	6 (0, 14)	10 (3, 16)	**<0.01** ^#^
Hospital days (median, IQR)	16 (10, 24)	15 (13, 19.5)	15 (12, 22)	0.63^#^
ICU days (median, IQR)	14 (9, 20)	13 (10, 16)	14 (10, 18)	0.27^#^
Percutaneous Endoscopic Gastrostomy placed?				0.72
Yes, *n* (%)	16 (20.3%)	7 (17.5%)	23 (19.3%)	
No	63 (79.8%)	33 (82.5%)	96 (80.7%)	
Tracheostomy placed?				0.14*
Yes, *n* (%)	14 (17.7%)	3 (7.7%)	17 (14.4%)	
No	65 (82.3%)	36 (92.3%)	101 (85.6%)	
Disposition				**<0.01** *
Death (%)	26 (32.9%)	3 (7.5%)	29 (24.4%)	
Home	26 (32.9%)	23 (57.5%)	49 (41.2%)	
Acute rehab	7 (8.9%)	6 (15.0%)	13 (10.9%)	
Other hospital	11 (13.9%)	2 (5.0%)	13 (10.9%)	
Nursing facility	9 (11.4%)	5 (12.5%)	14 (11.8%)	
Drug rehab	0 (0%)	1 (2.5%)	1 (0.8%)	
Poor composite outcome^a^				**<0.01**
Yes, *n* (%)	45 (57.0%)	12 (30.0%)	57 (47.9%)	
No	34 (43.0%)	28 (70.0%)	62 (52.1%)	

† *Statistically significant values are given in bold (p < 0.05)*.

a *Poor composite outcome is defined as incidence of percutaneous endoscopic gastrostomy, tracheostomy, discharge to a nursing facility, and/or hospital death*.

# *Wilcoxon Rank-sum test*.

* *Fisher's exact test*.

*DCI, delayed cerebral ischemia; IQR, interquartile range; EVD, external ventricular drain; ICU, intensive care unit*.

**Table 4:** Relative difference of major in-hospital outcomes based on admission glycemic gap ≥ 30 mg/dL, adjusted with co-variates.

**In-hospital outcomes**		**Covariate adjustment**	
		**Unadjusted**	**Adjusted for HH, mF, GCS on admission**
EVD days	Ratio* (95% CI)	1.69 (1.16, 2.48)	1.51 (1.04, 2.21)
	*p*-value^†^	**<0.01**	**0.03**
Hospital days	Ratio* (95% CI)	1.07 (0.85, 1.36)	1.05 (0.82, 1.35)
	*p*-value	0.55	0.68
Vasospasm	Ratio* (95% CI)	1.09 (0.80, 1.50)	1.12 (0.79, 1.57)
	*p*-value	0.58	0.52
DCI	Ratio* (95% CI)	1.23 (0.87, 1.74)	1.24 (0.85, 1.80)
	*p*-value	0.25	0.27
Poor composite outcome^#^	Ratio* (95% CI)	1.90 (1.00, 3.59)	1.07 (0.55, 2.09)
	*p*-value	0.05	0.85
In-hospital mortality	Ratio* (95% CI)	4.39 (1.33, 14.50)	2.10 (0.60, 7.30)
	*p*-value	**0.02**	0.24

* *Ratio of days for EVD days and hospital days, rate ratio or RR for vasospasm, DCI, poor composite outcome, and in-hospital mortality*.

† *Statistically significant values are given in bold (p < 0.05)*.

# *Poor composite outcome is defined as incidence of percutaneous endoscopic gastrostomy, tracheostomy, discharge to a nursing facility, and/or hospital death*.

*HH, Hunt and Hess scale; mF, modified Fisher scale; GCS, Glasgow Coma Scale; EVD, external ventricular drain; CI, confidence interval; DCI, delayed cerebral ischemia*.

### *Post-hoc* Power Analysis

The *post-hoc* power analysis showed the power of Poisson regression depended on the effect size of rate ratio and the overall association between aGG and covariates. With an over association of *r* = 0.5 between aGG and covariates (modified Fisher, Hunt and Hess, and GCS on admission), estimated from a logistic regression, an effect size of RR = 3.37 is required to reach 80% power. The Hunt and Hess grade especially had a strong association with both aGG and in-hospital mortality.

## Discussion

Glycemic gap is a standardized way to measure acute stress-induced hyperglycemia relative to baseline glycemic status. Obtaining aGG only requires a peripheral blood draw, and its analysis does not involve detailed clinical or radiologic evaluations. Our study describes the correlation between aGG and short-term outcomes in an adult population of aSAH. An elevated aGG ≥30 mg/dL was common in our cohort, occurring in 66.4% of all patients. This elevated status was associated with markers of disease severity and in-hospital outcomes, strengthening the concept that the entity is an indicator of physiologic stress response to aSAH. According to ROC curve analysis, aGG outperformed admission glucose in predicting in-hospital mortality and was similarly accurate in discerning poor composite outcome.

We were unable to demonstrate that aGG independently predicts in-hospital mortality and poor composite outcome in aSAH after controlling for Hunt and Hess scale, modified Fisher scale, and GCS. The unadjusted relative risks for poor composite outcome and in-hospital mortality halved when adjusted for the three clinical/radiographic severity measures associated with complicated hospital course and outcome in SAH. Despite not reaching statistical significance, the rate ratio of 2.10 (95% CI 0.60, 7.30) for in-hospital mortality in patients with aGG ≥30 mg/dL in our multivariable analysis suggests that our study is underpowered and that further study may be needed to determine the true association between aGG and the outcome. As expected, Hunt and Hess scale had a strong association with both aGG and with in-hospital mortality and was likely the primary driver in the elevated aGG group. Interestingly, there was no similar hint that aGG independently predicts poor composite outcome (RR = 1.07, 95% CI 0.55, 2.09), possibly because aGG specifically identifies acute pathophysiologic processes leading to the extreme outcome of death. Poor composite outcome, in comparison, encompasses non-fatal outcomes, and aGG may lose its association with less severe health consequence. Our multivariable analysis also suggests that aGG is independently associated with longer EVD duration. The ratio of days for EVD and for hospitalization changed minimally with adjustment, indicating that these in-hospital outcomes were not significantly mediated by the adjusted factors. Larger, prospective studies are needed to further elucidate the importance of aGG in patients with aSAH.

Based on the ROC curve analysis, aGG may be a superior predictor for in-hospital mortality in aSAH patients compared to admission glucose. Our cutoff value of 30 mg/dL was deemed optimal considering the clinical context under which this entity is used—to capture most deaths with a reasonable false positive rate. This is close to the cutoff of 26 mg/dL, derived through a ROC analysis in another study on aneurysmal and non-aneurysmal SAH (17). In comparison, one previous study in a medical ICU with 12.4% of the cohort having a primary neurologic condition, an aGG cutoff of 80 mg/dL was proposed in discerning mortality in diabetics (14). Considering the predictive role of aGG in inclusion of poor outcomes, the authors believed that the higher aGG cutoff of 80 mg/dL would be less ideal with a lower sensitivity; however, it would expectedly confer a higher specificity. More study is needed to evaluate whether a universally effective aGG cutoff for mortality exists for diabetics and non-diabetics.

From our univariate analysis, elevated aGG was associated with known patient history of diabetes mellitus, but not with HbA1c level on admission. One plausible explanation is that the chronic systemic inflammation, insulin resistance, and vasculopathy in diabetics can add to the vulnerability of patients during the acute illness to experience hyperglycemia. Further investigation, especially with a larger diabetic group, can help further elucidate the implication of HbA1c in diabetics, including in those with well-controlled, poorly controlled, or newly diagnosed disease.

Admission GG was also associated with higher WBC, lower bicarbonate, and higher lactate level, indicative of acute physiological stress reaction. Acute glucose derangement has been seen in aSAH in the setting of lactic acidemia (20), sepsis (3), coagulopathy (10), symptomatic cerebral vasospasm (21), and DCI (4). Our study shows that those with aGG ≥30 mg/dL had a higher proportion of DCI from Chi-squared test (Table 3) but no difference in vasospasm. One possible explanation is that there is an underlying microvasculature process leading to cerebral ischemia. Such a hypothesis has been suggested for patients with sepsis due to infectious etiologies (22) and could be a pathophysiological process for mitochondrial dysfunction in acute aSAH (23). Admission GG also may not be directly biologically causative of poor outcome after aSAH, but our results are hypothesis-generating.

Additionally, we found that more poor grade patients had aGG ≥30 mg/dL. One could postulate that such an association is a marker of disease severity (i.e., stress hyperglycemia). One could also theorize that the microcirculation as well as hyperacute vasospasm are also involved in the acute setting of poor grade patients. A previous case series demonstrated that hyperacute vasospasm could play a role in the outcome and mortality of poor grade aSAH patients (24). As was discussed in the previous paragraph, a combination of microcirculatory dysfunction and hyperacute vasospasm could play a role in defining patients that become poor grade aSAH. This two-hit hypothesis could further explain mitochondrial dysfunction in poor grade aSAH.

Our assessment of the prognostic value with aGG should be interpreted with caution. Our study supported a statistically significant trend of increased risk of DCI with elevated glycemic gap with Chi-squared test (Table 3). However, when tested the rate ratio using negative binomial model with and without covariates adjustment (Table 4), the association only remained a trend without statistical significance. Chi-squared test, similarly to odds ratio in a logistic regression model, can exaggerate an association when the outcome rate is high (25), as in our DCI occurrence of 29.4%. Therefore, the statistical significance diminished when tested with negative binomial model (Table 4). Since our sample size of 119 is not considered small, we can conclude that there is no large effect size for the association between DCI and aGG. A statistically significant rate ratio could be detected with a larger sample size. Furthermore, an aGG cutoff of at or above 30 mg/dL showed a non-significant trend of increased mortality with the RR of 2.10, and a greater sample size may have detected an independent difference. A threshold value of 30 mg/dL might serve as a clinically useful, general rule to help identify aSAH patients at highest risk for poor outcomes; however, the aGG value should be interpreted on an individual basis.

Our study is limited by the retrospective, observational design, which is subject to missing or misclassified data and unmeasured confounding. We cannot exclude a possibility that those with HbA1c ordered were more likely to be diabetic or have a higher disease severity, creating a potential selection bias; however, we routinely order HbA1c on patients admitted to our neuro ICU. We are also unable to confirm whether our patients received pre-treatment for hyperglycemia or routine insulin for diabetes mellitus prior to recorded glucose, which may have influenced admission glucose and calculated aGG levels. Variables such as socioeconomic or insurance status, or withdrawal of life-sustaining care may influence inpatient mortality and post-hospital disposition but were not captured in this analysis. Additionally, outcomes post-discharge could not be assessed; therefore, it is unknown if aGG has any predictive power on long-term functional outcomes. Our definition of poor outcome, used in other secondary database analysis (19, 26), identified patients requiring nursing care for activities of daily living in the short-term, but it may not accurately predict long-term dependence. Lastly, correlative relationships do not imply causation, and further randomized clinical trials would be needed to determine if active aGG control will lead to improved outcomes.

Our study confirms correlation between aGG and glucose, as well as between elevated aGG and several admission and inpatient factors known to be associated with hyperglycemia. We demonstrated that aGG was superior to admission glucose in predicting mortality, and that its level below 30 mg/dL served as a useful marker of significantly reduced mortality on our moderate-size cohort of critically ill aSAH patients. Further studies with a large size of diabetic patients are warranted to help better ascertain the mechanistic role of GG in the metabolic and inflammatory processes of aSAH as well as application in prognostication following aSAH with other known markers, as tested with the APACHE-II score in prediction of ICU mortality in general (14). Finally, an equally important area to study is to assess whether pursuing longitudinal glycemic control based on aGG is more effective and feasible.

## Conclusions

An elevated admission glycemic gap is common in aSAH patients and is associated with disease severity. In our study, an aGG ≥30 mg/dL was not independently associated with in-hospital mortality and poor outcome after controlling for disease severity, but the study was underpowered to find an independent association. Future study is needed to better understand the clinical significance of this marker and evaluate its use as a predictor of important clinical outcomes in this population.

## Data Availability Statement

The raw data supporting the conclusions of this article will be made available by the authors, without undue reservation.

## Ethics Statement

The studies involving human participants were reviewed and approved by University of Southern California Health Sciences Institutional Review Board. Written informed consent for participation was not required for this study in accordance with the national legislation and the institutional requirements.

## Author Contributions

PS, PN, SB, and MK-T: acquisition of data, analysis and interpretation of data, and drafting or revising the article. RP: acquisition of data, conception and design, analysis and interpretation of data, and drafting or revising the article. JM and SC: analysis and interpretation of data and drafting or revising the article. BE: conception and design, analysis and interpretation of data, and drafting or revising the article. All authors contributed to the article and approved the submitted version.

## Funding

This research was supported by an institutional award from the Southern California Clinical and Translational Science Institute (Grant Number: UL1TR001855).

## Conflict of Interest

The authors declare that the research was conducted in the absence of any commercial or financial relationships that could be construed as a potential conflict of interest.

## Publisher's Note

All claims expressed in this article are solely those of the authors and do not necessarily represent those of their affiliated organizations, or those of the publisher, the editors and the reviewers. Any product that may be evaluated in this article, or claim that may be made by its manufacturer, is not guaranteed or endorsed by the publisher.
